# From “Dark Matter” to “Star”: Insight Into the Regulation Mechanisms of Plant Functional Long Non-Coding RNAs

**DOI:** 10.3389/fpls.2021.650926

**Published:** 2021-06-07

**Authors:** Qingshuai Chen, Kui Liu, Ru Yu, Bailing Zhou, Pingping Huang, Zanxia Cao, Yaoqi Zhou, Jihua Wang

**Affiliations:** ^1^Shandong Provincial Key Laboratory of Biophysics, Institute of Biophysics, Dezhou University, Dezhou, China; ^2^Institute for Glycomics and School of Information and Communication Technology, Griffith University, Gold Coast, QLD, Australia; ^3^Institute for Systems and Physical Biology, Shenzhen Bay Laboratory, Shenzhen, China; ^4^Peking University Shenzhen Graduate School, Shenzhen, China

**Keywords:** plants, long non-coding RNAs, interaction, functional molecular, gene expression

## Abstract

Long non-coding RNAs (lncRNAs) play a vital role in a variety of biological functions in plant growth and development. In this study, we provided an overview of the molecular mechanisms of lncRNAs in interacting with other biomolecules with an emphasis on those lncRNAs validated only by low-throughput experiments. LncRNAs function through playing multiple roles, including sponger for sequestering RNA or DNA, guider or decoy for recruiting or hijacking transcription factors or peptides, and scaffold for binding with chromatin modification complexes, as well as precursor of microRNAs or small interfering RNAs. These regulatory roles have been validated in several plant species with a comprehensive list of 73 lncRNA–molecule interaction pairs in 16 plant species found so far, suggesting their commonality in the plant kingdom. Such initial findings of a small number of functional plant lncRNAs represent the beginning of what is to come as lncRNAs with unknown functions were found in orders of magnitude more than proteins.

## Introduction

Non-coding RNAs (ncRNAs), transcribed in over 90% of eukaryotic genomes of fungi, plants, and animals (Chekanova et al., 2007), were initially thought as “dark matter” in transcripts because their expressions are low, the sequences are poorly conserved, and the protein-coding potentials are absent (Palazzo and Koonin, 2020). With the advances in high-throughput sequencing and other experimental techniques, more ncRNAs begin to reveal their functional roles. In particular, they had been shown to play a key role in regulating the gene expressions at epigenetic, transcriptional, posttranscriptional, and posttranslational levels.

Long non-coding RNAs (lncRNAs) refer to those ncRNAs longer than 200 nucleotides (nt) in length (Rai et al., 2019; Palazzo and Koonin, 2020), as poorly conservative in sequence as other shorter ncRNAs (Chekanova, 2015). LncRNAs have been classified into three types according to their genomic locations: long intergenic ncRNAs (lincRNAs) in the intergenic regions, intronic ncRNAs (incRNAs) in the intronic regions, and natural antisense transcripts (NATs) from the antisense coding regions (Mattick and Rinn, 2015; Rai et al., 2019). Most lncRNAs are transcribed by RNA polymerase II (Pol II) or III (Pol III), and some could be produced by plant-specific RNA Pol IV and V (Wierzbicki et al., 2008). In this article, we summarized the molecular functions of plant lncRNAs discovered and validated by the low-throughput experiments with a specific focus on the relationship between lncRNAs and other biological macromolecules in regulating RNA activity, protein modification, and chromatin remodeling.

## Overview

**Figure 1:**
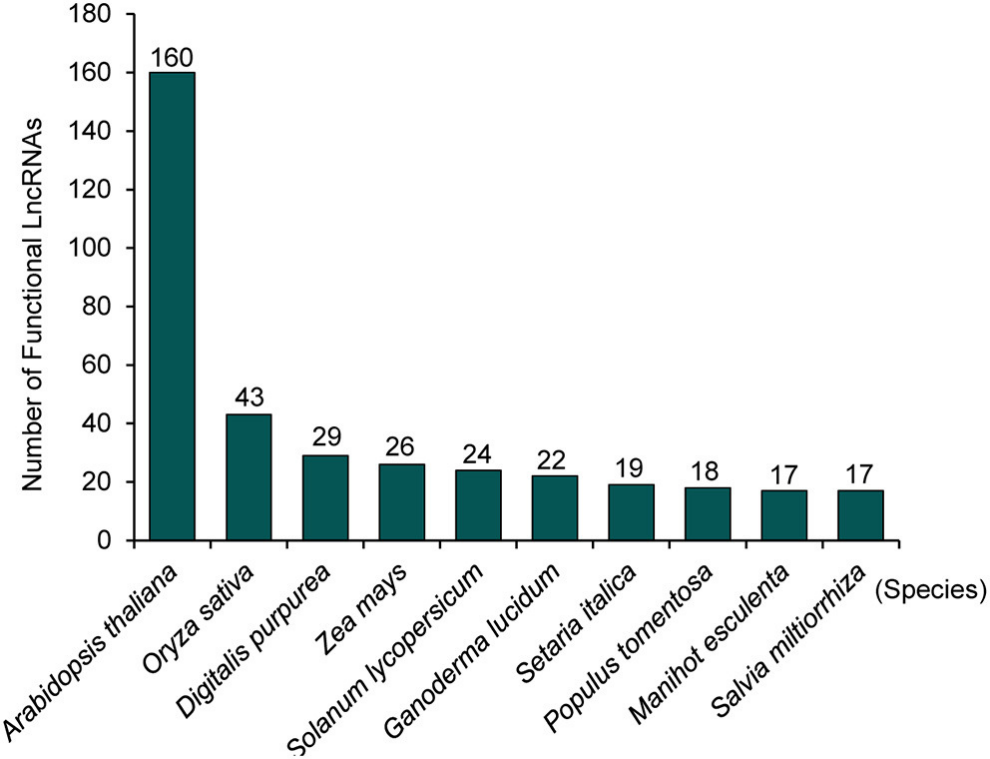
Statistics of the number of functional lncRNAs in top 10 plant species based on the experimentally validated functional lncRNAs in plants, according to EVLncRNA2.0 (Zhou et al., 2020).

**Figure 2:**
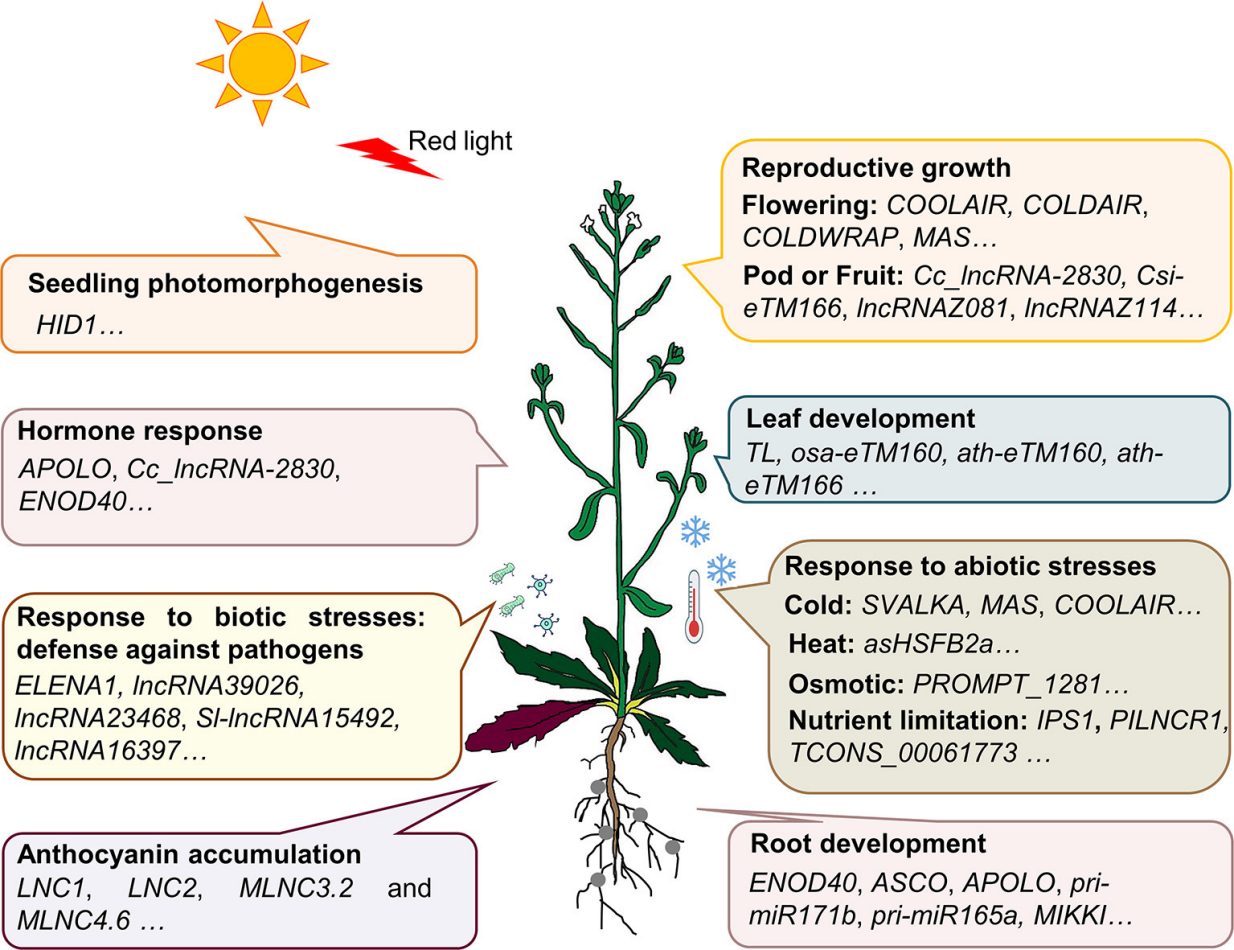
LncRNAs play extensive roles in most aspects of plant growth and development. During plant growth and development, multiple lncRNAs are validated to play crucial roles. *HID1* plays an active role in seedling photomorphogenesis under continuous red light; *APOLO, Cc_lncRNA-2830*, and *ENOD40* were demonstrated to respond to the auxin treatment; *ELENA1, lncRNA39026*, and *lncRNA23468* enhance resistance to pathogen attacks; *COOLAIR, COLDAIR, COLDWRAP*, and *MAS* respond to cold, and involve in regulating the flowering time in vernalization; *TL, osa-eTM160, ath-eTM160*, and *ath-eTM166* affect the leaf shape; *SVALKA* regulates cold signal transduction; *asHSFB2a* is induced by heat stress and affects grain yield; *IPS1* and *PILNCR1* are induced by phosphate deficiency; *TCONS_00061773* respond to nitrate signal; *LNC1, LNC2, MLNC3.2*, and *MLNC4.6* are involved in anthocyanin accumulation; *ENOD40, APOLO, pri-miR171b, pri-miR165a*, and *MIKKI* are involved in root nodule organogenesis or lateral root development.

A large number of lncRNAs in plants were found by using transcriptome sequencing and bioinformatics analysis (Muers, 2011; Li et al., 2014, 2019; Hou et al., 2017; Yu T. et al., 2019). Several studies have summarized the bioinformatics tools and database resources for plant lncRNA identification and prediction (Jha et al., 2020; Waseem et al., 2020). However, until present, only a small number of these lncRNAs have been validated by the low-throughput experimental analysis. For example, Plant Long Non-Coding RNA Database version 2.0 (Jin et al., 2020) has 1,246,372 lncRNAs and predicted lncRNA targets in 80 species, and NONCODEV6 contains 94,697 lncRNAs in 23 plant species (Zhao et al., 2020). According to the experimentally validated functional lncRNA (EVLncRNA) predictions, nearly 30% of lncRNAs obtained by high-throughput sequencing have biological functions (Zhou et al., 2019). If lncRNAs are calculated on the order of millions, there are more than 200,000 predicted functional lncRNAs based on predictions (Zhou et al., 2019). By comparison, only 506 functional lncRNAs in 56 plant species are curated in the database of EVLncRNAs2.0 (Zhou et al., 2020). The majority of these EVLncRNAs belong to the model plant *Arabidopsis thaliana* (Figure 1). As shown in Figure 2, lncRNAs were found to regulate the downstream gene expression in *cis* or *trans* and play crucial roles in bud dormancy (Li et al., 2020), flowering time (Heo and Sung, 2011; Wang Z. W. et al., 2014; Kim et al., 2017), seedling photomorphogenesis (Wang Y. et al., 2014), root organogenesis (Ariel et al., 2014), sexual reproduction (Fan et al., 2016), gene silencing (Huang et al., 2011), and response to biotic and abiotic stress (Wunderlich et al., 2014; Kindgren et al., 2018; Zhao et al., 2018). In other words, the regulatory role of lncRNAs is extensive in eukaryotes (Long et al., 2017). The low-throughput experimental techniques include RNA fluorescence *in situ* hybridization (FISH), Northern blot, real-time quantitative polymerase chain reaction (RT-qPCR), overexpression, RNA interference (RNAi), clustered regularly interspaced short palindromic repeat-associated protein 9 (CRISPR/Cas9), RNA pull-down, RNA immunoprecipitation (RIP), and chromatin immunoprecipitation (ChIP) (Wu et al., 2020). Table 1 provides the most up-to-date (November 30, 2020) list of all validated 73 lncRNA–molecule interaction pairs in 16 plant species that offer a glimpse of the molecular interaction network of functional lncRNAs in plant development and stress response. These interactions according to the interaction partners of lncRNAs are presented in the following sections.

**Table 1:** Functionally validated lncRNAs and their binding partners in plants.

**Name**	**Species**	**Interaction target**	**Level of interaction**	**Biological functions**	**References**
*IPS1*	*Arabidopsis thaliana*	miR399	RNA-RNA	Phosphate homeostasis	Franco-Zorrilla et al., 2007
*At4*	*Arabidopsis thaliana*	miR399	RNA-RNA	Phosphate homeostasis	Shin et al., 2006
*At4-1*	*Arabidopsis thaliana*	miR399	RNA-RNA	Phosphate homeostasis	Shin et al., 2006
*At4-2*	*Arabidopsis thaliana*	miR399	RNA-RNA	Phosphate homeostasis	Shin et al., 2006
*At4-3*	*Arabidopsis thaliana*	miR399	RNA-RNA	Phosphate homeostasis	Shin et al., 2006
*Mt4*	*Barrel clover*	miR399	RNA-RNA	Phosphate homeostasis	Shin et al., 2006
*TPSI1*	*Solanum lycopersicum*	miR399	RNA-RNA	Phosphate homeostasis	Liu et al., 1997
*PDIL1*	*Medicago truncatula*	miR399	RNA-RNA	Phosphate homeostasis	Wang et al., 2017
*PILNCR1*	*Zea mays*	miR399	RNA-RNA	Phosphate homeostasis	Du et al., 2018
*HvIPS1*	*Hordeum vulgare*	HvmiR399	RNA-RNA	Phosphate homeostasis	Huang et al., 2011
*HvIPS2*	*Hordeum vulgare*	HvmiR399	RNA-RNA	Phosphate homeostasis	Huang et al., 2011
*lncRNA39026*	*Lycopersicon esculentum*	miR-168a	RNA-RNA	Disease resistance	Hou et al., 2020
*lncRNA23468*	*Solanum lycopersicum*	miR-482b	RNA-RNA	Disease resistance	Jiang et al., 2019
*LNC1*	*Hippophae rhamnoides*	miR156a	RNA-RNA	Anthocyanin accumulation	Zhang et al., 2018
*LNC2*	*Hippophae rhamnoides*	miR828a	RNA-RNA	Anthocyanin accumulation	Zhang et al., 2018
*MLNC3.2*	*Malus domestica*	miRNA156a	RNA-RNA	Anthocyanin accumulation	Yang et al., 2019
*MLNC4.6*	*Malus domestica*	miRNA156a	RNA-RNA	Anthocyanin accumulation	Yang et al., 2019
*csi-eTM166*	*Citrus sinensis*	csi-miR166c	RNA-RNA	Fruit ripening	Ke et al., 2019
*LTCONS_00026271*	*Camellia sinensis*	novel_miR44	RNA-RNA	JA/MeJA synthesis and response	Zhu et al., 2019
*LTCONS_00020084*	*Camellia sinensis*	miR169d-5p_1	RNA-RNA	JA/MeJA synthesis and response	Zhu et al., 2019
*Cc_lncRNA-2830*	*Cajanus cajan*	miR-160h	RNA-RNA	Seed and pod development	Das et al., 2019
*MIKKI*	*Oryza sativa*	miR171	RNA-RNA	Root development	Cho and Paszkowski, 2017
*eTM160*	*Arabidopsis thaliana*	miR160	RNA-RNA	Leaf shape	Wu et al., 2013
*eTM166*	*Arabidopsis thaliana*	miR166	RNA-RNA	Leaf shape	Wu et al., 2013
*Cs1 g09600*	*Citrus sinensis*	miR-3954	RNA-RNA	Flowering time	Liu et al., 2017
*Cs1 g09635*	*Citrus sinensis*	miR-3954	RNA-RNA	Flowering time	Liu et al., 2017
*MuLnc1*	*Morus multicaulis*	miR-3954	RNA-RNA	Flowering time	Gai et al., 2018
*PMS1T*	*Oryza sativa*	miR2118	RNA-RNA	Male sterility	Fan et al., 2016; Komiya, 2017
*MSPPL1*	*Oryza sativa*	miR2118	RNA-RNA	Male sterility, meiosis	Zhang et al., 2020
*MSPPL2*	*Oryza sativa*	miR2118	RNA-RNA	Male sterility, meiosis	Zhang et al., 2020
*lncRNA15492*	*Solanum lycopersicum*	Sl-miR482a	RNA-RNA	Disease resistance	Jiang et al., 2020
*lncRNAZ081*	*Solanum lycopersicum*	miRNA6027	RNA-RNA	Ethylene response	Wang et al., 2018a
*lncRNAZ114*	*Solanum lycopersicum*	miRNA1919b	RNA-RNA	Ethylene response	Wang et al., 2018a
*lncRNAZ114*	*Solanum lycopersicum*	miRNA1919c	RNA-RNA	Ethylene response	Wang et al., 2018a
*TCONS_00061773*	*Solanum lycopersicum*	ptc-miR1448	RNA-RNA	Nitrogen deficient response	Chen et al., 2016
*TCONS_00061773*	*Solanum lycopersicum*	ptc-miR482a	RNA-RNA	Nitrogen deficient response	Chen et al., 2016
*TL*	*Oryza sativa*	*OsMYB60*	RNA-RNA	Leaf shape	Liu et al., 2018
*asHSFB2a*	*Arabidopsis thaliana*	*HSFB2a*	RNA-RNA	Heat stress response, gametophyte development	Wunderlich et al., 2014
*lncRNA16397*	*Solanum lycopersicum*	*SlGRX21*	RNA-RNA	Disease resistance	Cui et al., 2017
*lncRNA16397*	*Solanum lycopersicum*	*SlGRX22*	RNA-RNA	Disease resistance	Cui et al., 2017
*HID1*	*Arabidopsis thaliana*	*PIF3*	RNA-DNA	Seedling photomorphogenesis	Wang Y. et al., 2014
*MAS*	*Arabidopsis thaliana*	*MAF4*	RNA-DNA	Vernalization flowering	Zhao et al., 2018
*APOLO*	*Arabidopsis thaliana*	*PID*	RNA-DNA	Auxin response; lateral root development	Ariel et al., 2014
*APOLO*	*Arabidopsis thaliana*	*WAG2*	RNA-DNA	Auxin response; lateral root development	Mas and Huarte, 2020
*APOLO*	*Arabidopsis thaliana*	*AZG2*	RNA-DNA	Auxin response; lateral root development	Mas and Huarte, 2020
*LAIR*	*Oryza sativa*	*LRK1*	RNA-DNA	Rice grain yield	Wang et al., 2018b
*SVALKA*	*Arabidopsis thaliana*	*CBF1*	RNA-DNA	Cold acclimation	Kindgren et al., 2018
*APOLO*	*Arabidopsis thaliana*	LHP1	RNA-Protein	Auxin response; lateral root development	Ariel et al., 2014
*COOLAIR*	*Arabidopsis thaliana*	FCA	RNA-Protein	Vernalization flowering	Tian et al., 2019
*COLDAIR*	*Arabidopsis thaliana*	PRC2	RNA-Protein	Vernalization flowering	Heo and Sung, 2011
*COLDWRAP*	*Arabidopsis thaliana*	PRC2 (CLF)	RNA-Protein	Vernalization flowering	Kim and Sung, 2017
*ASL*	*Arabidopsis thaliana*	AtRRP6L1	RNA-Protein	Flowering	Shin and Chekanova, 2014
*MAS*	*Arabidopsis thaliana*	MAS-WDR5a	RNA-protein	Vernalization flowering	Zhao et al., 2018
*LAIR*	*Oryza sativa*	OsMOF	RNA-Protein	Rice grain yield	Wang et al., 2018b
*LAIR*	*Oryza sativa*	OsWDR5	RNA-Protein	Rice grain yield	Wang et al., 2018b
*ELENA1*	*Arabidopsis thaliana*	MED19a	RNA-Protein	Disease resistance	Seo et al., 2017
*ELENA1*	*Arabidopsis thaliana*	FIB2	RNA-Protein	Disease resistance	Seo et al., 2019
*ENOD40*	*Medicago truncatula*	MtRBP1 (MtNSR1)	RNA-Protein	Nuclear-cytoplasmic relocalization, root nodules formation	Campalans et al., 2004
*ENOD40*	*Medicago truncatula*	AtNSRa	RNA-Protein	Nuclear-cytoplasmic relocalization	Bardou et al., 2014
*ENOD40*	*Medicago truncatula*	AtNSRb	RNA-Protein	Nuclear-cytoplasmic relocalization	Bardou et al., 2014
*ASCO/lnc351*	*Arabidopsis thaliana*	AtNSRa	RNA-Protein	Alternative splicing, root development	Bardou et al., 2014
*ASCO/lnc351*	*Arabidopsis thaliana*	AtNSRb	RNA-Protein	Alternative splicing, root development	Bardou et al., 2014
*ASCO/lnc351*	*Arabidopsis thaliana*	PRP8a	RNA-Protein	Alternative splicing, root development	Rigo et al., 2020
*ASCO/lnc351*	*Arabidopsis thaliana*	SmD1b	RNA-Protein	Alternative splicing, root development	Rigo et al., 2020
*PROMPT_1281*	*Populus simonii*	MYB	RNA-Protein	Osmatic stress response	Song Y. et al., 2019
*MtENOD40*	*Medicago truncatula*	MtSNARP1	RNA-peptide	Root nodules formation	Laporte et al., 2010
*MtENOD40*	*Medicago truncatula*	MtSNARP2	RNA-peptide	Root nodules formation	Laporte et al., 2010
*GmENOD40*	*Glycine max*	nodulin100	peptide A-Protein	Root nodules formation	Rohrig et al., 2002
*GmENOD40*	*Glycine max*	nodulin100	peptide B-Protein	Root nodules formation	Rohrig et al., 2002
*pri-miR165a*	*Arabidopsis thaliana*	miPEP165a	LncRNA encodes peptide	Root development	Lauressergues et al., 2015
*pri-miR171b*	*Medicago truncatula*	miPEP171b	LncRNA encodes peptide	Root development	Lauressergues et al., 2015
*pri-miR858a*	*Arabidopsis thaliana*	miPEP858a	LncRNA encodes peptide	Flavonoid biosynthesis	Sharma et al., 2020
*pri-miR171d*	*Vitis vinifera*	miPEP171d1	LncRNA encodes peptide	Adventitious root formation	Chen et al., 2020

## Long Non-Coding RNA–RNA Relations

### Long Non-Coding RNA as an Endogenous Target Mimic to Repress microRNAs

**Figure 3:**
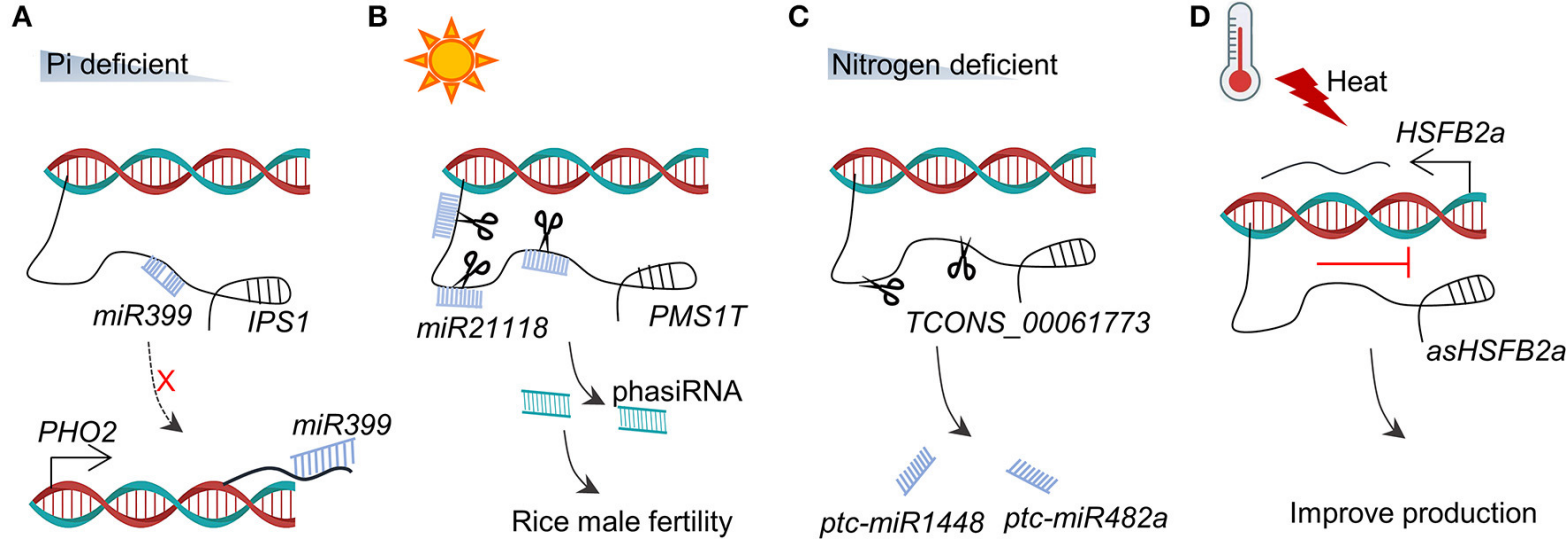
The relationship between lncRNAs and RNAs. **(A)** Under Pi deficiency, *IPS1* serves as an eTM to bind with *miR399*, and prevents the interaction between *miR399* and *PHO2* mRNA. **(B)** *PMS1T* is sensitive to photoperiod and is targeted by *miR2118* to produce phasiRNA. **(C)** *TCONS_00061773* acts as the precursor of *ptc-miR1448* and *ptc-miR482a* in response to nitrogen deficiency. **(D)** Heat stress induces *asHSFB2a* and then represses *HSFB2a*.

The association between lncRNAs and small RNAs is perhaps the most reported relationship in plants. MicroRNAs (miRNAs) that are small ncRNAs (21–23 nt in length) play vital roles in diverse biological processes such as root development (Bazin et al., 2012), vegetative-to-reproductive transition (Yang et al., 2013), formation of phytohormones, and biotic/abiotic stress response (Yamamuro et al., 2016; Song X. et al., 2019). In principle, lncRNA could work as a “sponger” to adsorb a complementary miRNA to indirectly regulate the target genes of the miRNA. These lncRNAs are known as competitive endogenous RNAs (ceRNAs) or “endogenous target mimic (eTM).” In *Arabidopsis*, the lncRNA *IPS1* (*INDUCED BY PHOSPHATE STARVATION1*) is complementary to miR399, but it contains a mismatched loop to interrupt the pairing at the miRNA cleavage site (Franco-Zorrilla et al., 2007). As illustrated in Figure 3A, under the phosphate (Pi) starvation condition, *IPS1* is induced to sequester miR399. Sequestration of miR399 increases the transcriptional level of *miR399* target *PHO2* (encoding an E2 ubiquitin conjugase-related enzyme), which subsequently reduces the Pi content of the shoot (Franco-Zorrilla et al., 2007). The family members of *IPS1* have been identified in several species with the same functional mechanism as the eTM of miR399s, including *At4, At4-1, AT4-2*, and *AT4-3* in *A. thaliana* (Shin et al., 2006), *TPSI1* in *Solanum lycopersicum* (Liu et al., 1997), *Mt4* in barrel clover (Burleigh and Harrison, 1999), *PILNCR1* in maize (Du et al., 2018), *PDIL1* in *Medicago truncatula* (Wang et al., 2017), and *HvIPS1* and *HvIPS2* in *Hordeum vulgare* (Huang et al., 2011) (Table 1).

The above-mentioned eTM regulatory mechanism that occurs between other lncRNAs and miRNAs is conserved in different plant species and in biological pathway. As shown in Table 1, lncRNAs act as ceRNAs to sequestrate the silencing of miRNA for target genes, such as regulating leaf shapes in *Arabidopsis* (Wu et al., 2013), altering anthocyanin contents in *Hippophae rhamnoides* (Zhang et al., 2018), affecting the citrus fruit development (Ke et al., 2019), responding to auxin signal (Das et al., 2019), promoting jasmonic acid (JA) and methyl jasmonate (MeJA) biosynthesis and signal transduction pathways (Zhu et al., 2019), regulating rice root development (Cho and Paszkowski, 2017), and enhancing resistance to disease (Jiang et al., 2019; Hou et al., 2020).

### Long Non-Coding RNA as a Precursor of phasiRNAs

The long non-coding RNAs that are binding with the complementary miRNA may lead to their cleavage to yield phased small-interfering RNAs (phasiRNAs). In eukaryote, lncRNAs or other RNAs are complemented and cleaved by miRNA to generate single-stranded RNAs first, and then to form double-stranded RNAs from the lysed RNA *via* RNA-dependent RNA polymerase, which are further processed into phasiRNA of 21–24 nt. Finally, phasiRNAs were loaded into AGONAUTE proteins for silencing RNA (Komiya, 2017). As shown in Figure 3B, the lncRNA *PMS1T*, encoded by *photoperiod-sensitive genic male sterility 1* (*Pms1*) locus in rice, is cleaved by miR2118 to generate 21-nt phasiRNAs. The abundance of phasiRNAs is associated with the photoperiod-sensitive male sterile (Fan et al., 2016; Komiya, 2017). Two other anther-specifically expressed lncRNAs, *Male Sterility-related PhasiRNA Precursor LincRNAs1* (*MSPPL1*) and *MSPPL2*, are also cleaved by miR2118 to yield phasiRNAs that play a role in the posttranscriptional regulation during meiosis in rice (Zhang et al., 2020). This mechanism is well-known in different plant development processes (Table 1); among these, *Cs1g09600* and *Cs1g09635* are cleaved by miR3954 to yield phasiRNAs, playing a role in the regulation of flowering in *Citrus sinensis* (Liu et al., 2017), *MuLnc1* is cleaved by miR3954 to produce phasiRNAs, disrupting the expression of the calmodulin-like protein gene *CML27* in *Morus multicaulis* (Gai et al., 2018), and *Sl-lncRNA15492*-miR482a-phasiRNAs affect the resistance to *Phytophthora infestans* in tomato (Jiang et al., 2020).

### Long Non-Coding RNA as a Precursor of miRNA

In plants, the biogenesis of miRNAs is precisely controlled (Yu Y. et al., 2019). The production of miRNA undergoes a complex process in which *MIR* genes mainly located in the intergenic regions are first transcribed into primary miRNAs, then modified into the precursor miRNAs, forming miRNA duplexes, and finally forming mature miRNA to guide the posttranscriptional gene silencing (Sanei and Chen, 2015). Some lncRNAs act as the precursors of mature miRNAs (Liu et al., 2015; Yu Y. et al., 2019). In *Arabidopsis*, two lncRNAs, *npc83*, and *npc521*, are found to be the miRNA precursors for *MIR86lncRNA9A* and *MIR160C*, respectively (Ben Amor et al., 2009). The same phenomena are found in other plant species. *TCONS_00061773* are the precursors of ptc-miR1448 and ptc-miR482a, which involve in the defense mechanism to prevent nitrogen deficiency in *S. lycopersicum* (Chen et al., 2016) (Figure 3C). In tomato fruit, *lncRNAZ081* is the precursor of miRNA6027, and *lncRNAZ114* is the precursor of miRNA1919b and miRNA1919c (Wang et al., 2018a). In this study, we described only miRNA precursors for those lncRNAs that were validated by the low-throughput experiments.

### Long Non-Coding RNAs as Antisense Transcripts Co-Expressed With mRNAs

Some lncRNAs can be produced as antisense transcripts in the coding regions, and *cis*-regulate mRNA transcript through indirect physical interaction. As shown in Figure 3D, *asHSFB2a* in *A. thaliana* is a natural long non-coding antisense RNA of *HSFB2a* induced by heat stress (Wunderlich et al., 2014). The overexpressed *asHSFB2a* leads to an interrupt of *HSFB2a*, which subsequently improves the production in vegetative development (Wunderlich et al., 2014). Antisense lncRNAs were also found in *S. lycopersicum* and *Oryza sativa*. The antisense transcript of *SlGRX22, lncRNA16397*, upregulates the *SlGRX22* and *SlGRX21* expression and enhances the disease resistance of *P. infestans* by tomato (Cui et al., 2017). *TWISTED LEAF* (*TL*) in rice is an antisense transcript of R2R3-MYB transcription factor gene, namely, *MYB60* locus. The RNA interference and the overexpression experiments indicate that *TL cis*-suppresses the *MYB60* expression in regulating leaf blade flattening in rice by altering the chromatin structure (Liu et al., 2018).

## Long Non-Coding RNA–DNA Interactions

### Long Non-Coding RNA–DNA Binding for Modulating Chromatin Loop Dynamics

**Figure 4:**
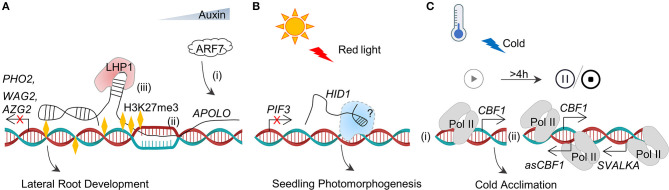
LncRNA–DNA interactions. **(A)** (i) Upregulation of *APOLO* by ARF7 after auxin treatment, (ii) direct binding between *APOLO* and auxin-responsive genes chromatin through base complementarity to form R-loop in *cis* and *trans*. Expressions of these auxin-responsive genes contribute to the lateral development, and (iii) recruitment of LHP1 by *APOLO* to deposit H3K27me3 for closing the loop and then for silencing the *APOLO* target. **(B)** *HID1* binds to the proximal promoter of *PIF3* and represses its expression to modulate seedling photomorphogenesis. *HID1* may function by combining with proteins although the detail is still unknown. **(C)** (i) Sense *CBF1* is initiated by cold exposure, and the expression peaks after 4 h. (ii) Contemporaneously, *SVALKA* is induced in the antisense region of *CBF1*. RNA Pol II read-through transcription of *SVALKA* leads to the expression of *asCBF1* and an increase of RNA Pol II occupancy on strands. The collision of RNA Pol II stalls *CBF1* sense transcription.

Dynamic chromatin topology is closely associated with the gene expression patterns. In *Arabidopsis*, the lncRNA *AUXIN-REGULATED PROMOTER LOOP* (*APOLO*) was shown essential for the lateral root development by regulating the transcription level of auxin-responsive genes. *APOLO* controls these auxin-responsive genes in *cis* or *trans* by modulating chromatin loops (Mas and Huarte, 2020; Figure 4A). *APOLO* is activated by AUXIN RESPONSE FACTORS7 (ARF7) after treating with auxin and *cis*-activates the neighboring gene *PID* through formatting a chromatin loop with the promoter of *PID* by reducing H3K27me3 in the loop (Ariel et al., 2014). The opened loop is closed after *APOLO* generated from RNA Pol II further recruits polycomb repressive complex1 (PRC1) to deposit the DNA methylation on the genomic region of *APOLO*-*PID* (Ariel et al., 2014). *APOLO* transcripts can also regulate several distant target genes such as *WAG2* and *AZG2* through forming R-loops in *trans* (Ariel et al., 2020). *APOLO* recognizes its distant target genes by base complementarity through the formation of DNA–RNA duplexes. Two complementary TTCTTC boxes in *APOLO* RNA are confirmed to be essential for the formation of *APOLO-*target DNA loops (Ariel et al., 2020).

### Long Non-Coding RNA–DNA Binding for Modulating Gene Transcriptions

The long non-coding RNAs can interact with DNA to regulate gene transcriptions. The knockdown of *HIDDEN TREASURE1* (*HID1*) (Wang Y. et al., 2014) in *Arabidopsis* displays elongated hypocotyls in continuous red light. ChIP-qPCR experiments revealed that *HID1* directly binds to the chromatin of the first intron of *PHYTOCHROME-INTERACTING FACTOR* 3 (*PIF3*) to repress its transcription in *trans* (Wang Y. et al., 2014). *HID1* is detected in nuclear. However, its protein partner is still unknown (Figure 4B). It should be noted that the sequence and secondary structure of *HID1* are highly conserved from moss to *Arabidopsis*, with a similar function in seedling photomorphogenesis (Wang Y. et al., 2014).

### Transcription of Long Non-Coding RNA Modulates DNA Transcription

In plants, a novel transcriptional regulation mechanism of coding gene depends on the transcription of the adjacent lncRNA (Kindgren et al., 2018). The lncRNA *SVALKA*, found in the cold-sensitive region of the *Arabidopsis* genome, is the antisense transcript between *C-repeat/dehydration-responsive element binding factor3* (*CBF3*) and *CBF1*. RNA Pol II read-through transcription of *SVALKA* leads to the expression of antisense *CBF1* (*asCBF1*), and the cascade of *SVALKA*-*asCBF1* represses *CBF1* transcribing through RNA Pol II collision stemming, leading to maximize the low-temperature tolerance of plants with minimal adaptation changes (Kindgren et al., 2018) (Figure 4C).

## Long Non-Coding RNA–Protein Interactions

### Long Non-Coding RNAs as Guides for Recruiting Proteins

**Figure 5:**
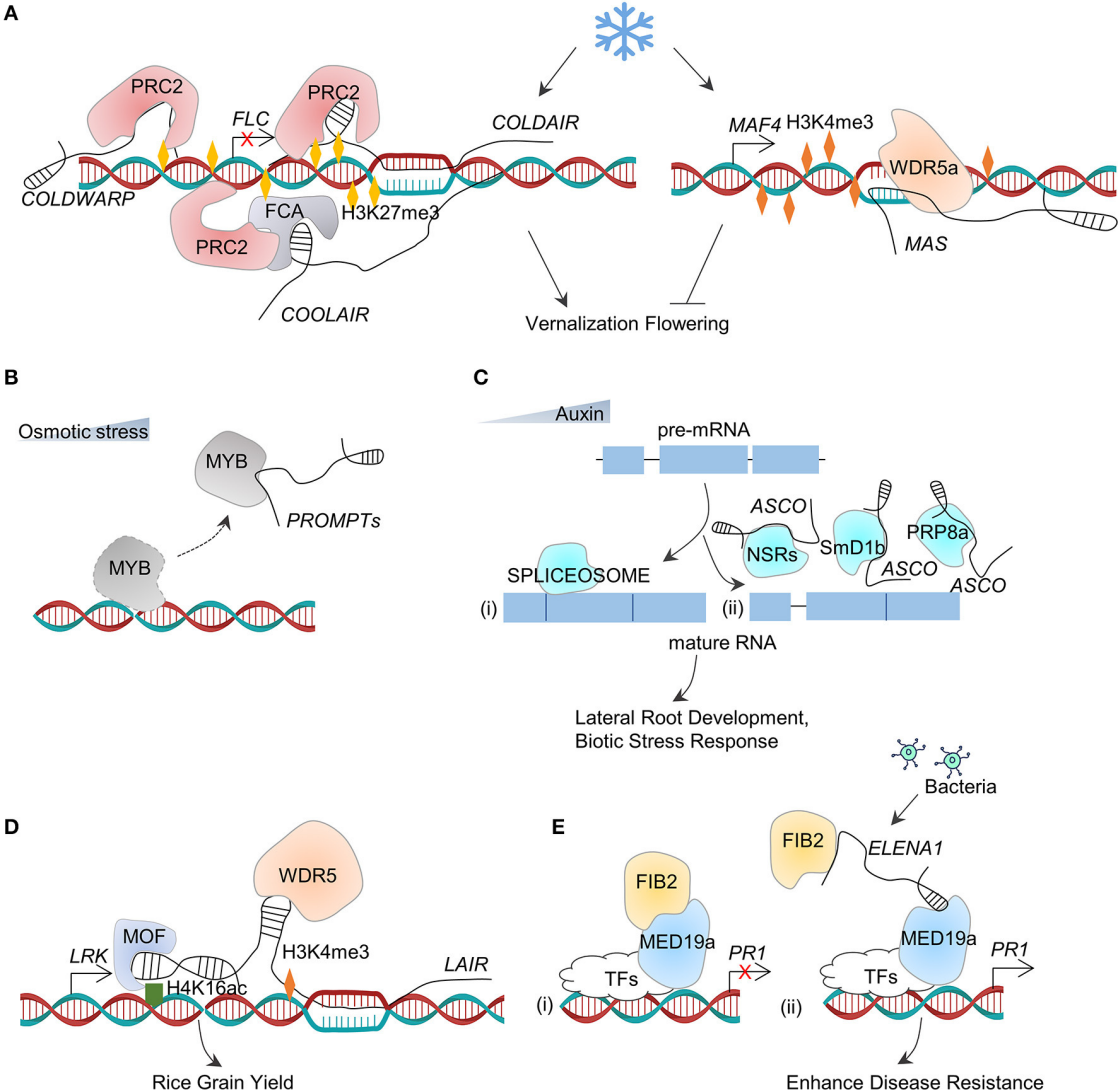
LncRNA–protein interactions. **(A)** During vernalization, cold temperature induces *COLDAIR, COLDWARP*, and *COOLAIR*. Then*, COLDAIR* and *COLDWARP* recruit PRC2 to enrich H3K27me3 in the regulation region of *FLC* to repress its expression, whereas *COOLAIR* directly binds with FCA to recruit PCR2 and deposit H3K27me3 in the chromatin of *FLC*. At the same time, cold signal induces the expression of *MAS* and then recruits WDR5a to deposit H3K4me3 in the chromatin of *MAF4* to increase its expression. Downregulation of *FLC* and upregulation of *MAF4* ensure the accurate flowering time during vernalization. **(B)** Under osmotic stress, *PROMPTs* act as decoys to hijack the MYB transcription factors (TFs) for preventing their regular functions as TFs in regulating gene transcription. **(C)** During alternative splicing of pre-mRNA, multiple splicing factors including NSRs, SmD1b, and PRP8a binds (ii) or not (i) to *ASCO*, together with pre-mRNA to modulate transcriptome diversity dynamically. **(D)** *LAIR*, derived from the antisense transcript of *LRK*, directly interacts with the genomic region of *LRK* and then acts as a scaffold to recruit MOF and WDR5 to deposit H4K16ac and H3K4me3, respectively. This leads to the upregulation of *LRK* expression and increase of grain yield. **(E)** (i) In the absence of pathogen (bacteria) treatment, FIB2 directly interacts with MED19a, and this complex represses *PR1* expression; (ii) after the pathogen treatment, the transcription level of lncRNA *ELENA1* increases and then binds to FIB2 and MED19a. After FIB2 dissociates, MED19a continues to bind on the promoter of *PR1* and activate its expression.

The long non-coding RNAs regulate the expression of genes by interacting with RNA-binding proteins. As mentioned earlier, after binding to the complementary DNA to form an R-loop, *APOLO* acts as a guide to recruit PRC1 component LIKE HETEROCHROMATIC PROTEIN1 (LHP1) to deposit the repressive histone marks (histone H3 Lys 27 trimethylation, H3K27me3) at the target chromatin and to repress the auxin-responsive gene transcription (Ariel et al., 2014; Figure 4A). The polycomb complex is recruited by lncRNAs during the process of vernalization-triggered flowering (Questa et al., 2016). The vernalization also induces three lncRNAs, such as *COLD INDUCED LONG ANTISENSE INTRAGENIC RNAs* (*COOLAIR*) (Baurle and Dean, 2006), *COLDWRAP* (Kim and Sung, 2017), and *COLD ASSISTED INTRONIC NON-CODING RNA* (*COLDAIR*) (Heo and Sung, 2011) which are derived from the antisense strand, the repressed promoter, and the first intron of *FLOWERING LOCUS C* (*FLC*), respectively (Figure 5A). These three lncRNAs involve in the deposition of H3K27me3 at *FLC* chromatin to repress *FLC* transcription (Kim and Sung, 2017). *COLDAIR* and *COLDWRAP* directly interact with PRC2, which catalyzes H3K27me3 (Heo and Sung, 2011; Kim and Sung, 2017; Kim et al., 2017), whereas *COOLAIR* binds to the protein FCA, which interacts with PRC2 subunit CURLY LEAF (CLF) to enrich H3K27me3 at *FLC* chromatin (Figure 5A). The protein phosphatase SSU72 physically interacts with FCA to antagonize the FCA binding with *COOLAIR* (Tian et al., 2019). In particular, an antisense lncRNA that derived by the same promoter of *COOLAIR* is independent on the vernalization in early-flowering *Arabidopsis* ecotypes. *ASL1* transcript physically associates with nuclear exosome component AtRRP6L1 (Callahan and Butler, 2008), maintaining H3K27me3 levels for silencing *FLC* (Shin and Chekanova, 2014).

Another cold-induced lncRNA is *MAS* that plays a regulatory role in the flowering time (Zhao et al., 2018). *MAS* is an NAT of the *MADS AFFECTING FLOWERING4* (*MAF4*) gene discovered in *Arabidopsis*, and it activates *MAF4* transcription involved in H3K4me3 deposition. Further assays showed that *MAS* binds to the locus of *MAF4* directly and recruits the COMPASS-like complexes (component WD repeat domain 5a, WDR5a) to enhance H3K4me3. The cold-induced formation of the MAS–WAR5a complex suppresses the premature flowering in vernalization (Zhao et al., 2018; Figure 5A). The interaction between lncRNAs and chromatin proteins modulates the chromatin 3D conformations of the neighboring or distant locus and affects the transcriptional activity in *cis* or *trans* (Figures 4A, 5A).

### Long Non-Coding RNAs as Decoys for Hijacking Proteins

*PROMPT_1281* is a promoter upstream transcript (PROMPT) lncRNAs, which is sensitive to osmotic stress in *Populus simonii*, through binding with MYBs transcription factors to interrupt them from interacting with DNA and lead to an increase in the expression of target genes in *cis* and *trans* (Song Y. et al., 2019; Figure 5B).

In *M. truncatula*, an lncRNA named as *MtENOD40* has a short open reading frame (sORF) and is important in root nodule organogenesis (Campalans et al., 2004). The functional analysis indicates that *MtENOD40* can interact with *M. truncatula* RNA-binding protein 1 (MtRBP1, also named as *M. truncatula* Nuclear Speckles RNA-binding protein 1, MtNSR1). When *MtENOD40* expressed in root tissues, the *ENOD40*-MtRBP1 complex would relocate from the nucleus to the cytoplasm (Campalans et al., 2004). Interestingly, the *Arabidopsis* orthologs of MtRBP1 (i.e., AtNSRa and AtNSRb), when co-expressed with *MtENOD40*, also lead to the relocation of AtNSRs from the nucleus to the cytoplasm, indicating the conserved relocation activity of *MtENOD40* (Bardou et al., 2014). The above-mentioned AtNSR proteins participate in alternative splicing (AS) of pre-mRNA in *Arabidopsis* (Bardou et al., 2014). *Lnc351*, also called as *AS competitor long-non-coding RNA* (*ASCO-lncRNA, ASCO*), is induced by auxin. This lncRNA would competitively bind AtNSRs with AS targets *in vitro* and *in vivo*, regulating transcriptome diversity in the formation of lateral root development (Bardou et al., 2014). Additionally, *ASCO* binds with multiple splicing factors including PRP8a and SmD1b and modulates the spliced genes in response to bacterial flagellin (Rigo et al., 2020) (Figure 5C). *ASCO* acts as an integrator through interacting with the spliceosome to dynamically modulate the transcriptome diversity.

### Long Non-Coding RNAs as Scaffolds for Linking Multiple Proteins Together

The long non-coding RNAs can act as scaffolds to link multiple proteins together for forming the functional ribonucleoprotein complexes. The lncRNA *LAIR* (*LRK* Antisense Intergenic RNA) is derived from the antisense strand of neighboring gene *LRK* (leucine-rich repeat receptor kinase) cluster. Its overexpression leads to the upregulated expression of *LRK* genes and increased rice grain yields (Wang et al., 2018b). It was found that *LAIR* directly binds to the genomic regions of *LRK1* and functions as a scaffold to link histone modification proteins such as Males-absent-on-the-first (OsMOF) and OsWDR5 *in vivo*, which in turn gives a rise in the enrichment of H3K4me3 and H4K16 acetylation (H4K16ac) at chromatin to active *LRK1* (Wang et al., 2018b; Figure 5D).

Similarly, as a part of plant immune response, the lncRNA *ELF18-INDUCED LONG NON-CODING RNA 1* (*ELENA1*) activates *PATHOGENESIS-RELATED GENE1* (*PR1*) by directly binding with Mediator subunit 19a (MED19a) and negative transcription regulator FIBRILLARIN2 (FIB2) to enrich *MED19a* on *PR1* promoter (Seo et al., 2017). More specifically, an association of *ELENA1* with FIB2 (Seo et al., 2019) hinders the inhibitory effect of FIB2 on *PR1* and allows the accumulation of more MED19a on *PR1* promoter to activate the gene expression (Figure 5E).

## Long Non-Coding RNA–Peptide Relations

### Long Non-Coding RNA–Peptide Interactions

**Figure 6:**
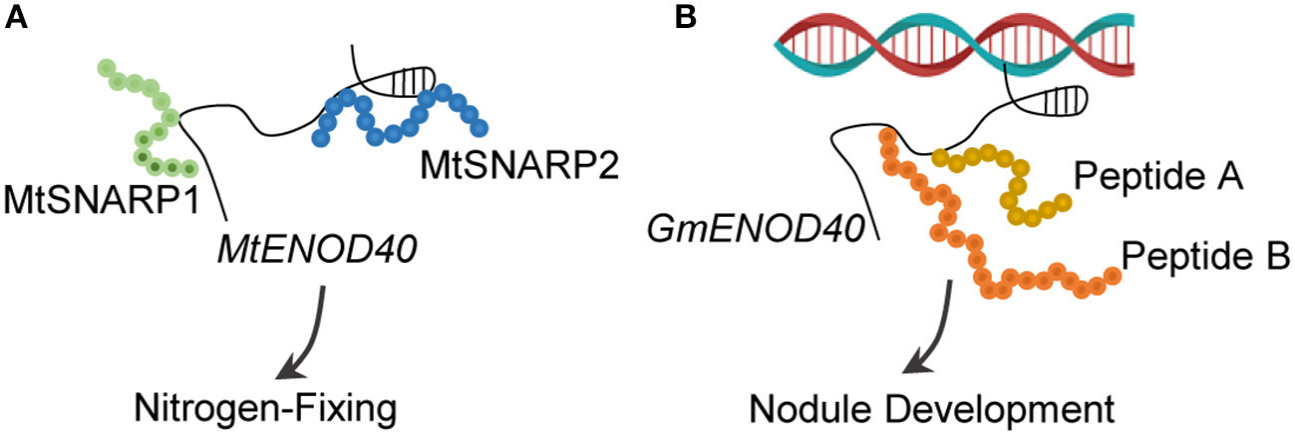
LncRNA–peptide relations. **(A)** *MtENOD40* acts as a decoy to titrate two small peptides, namely, MtSNARP1 and MtSNARP2. Both peptides play a key role in establishing nitrogen-fixing symbiosis between *Medicago truncatula* and *Sinorhizobium meliloti*. **(B)** *GmENOD40* contains sORFs that encode two small peptides (Peptide A and Peptide B) of 12 and 24 amino acids each. Both peptides involve in the development of nitrogen-fixing nodules.

In addition to interacting with RNA-binding proteins, *MtENOD40* was found to bind and repress two small nodulins, namely, Small Nodulin Acidic RNA-Binding Protein1 (MtSNARP1) and MtSNARP2 (Laporte et al., 2010). SNARPs are acidic peptides and are involved in the nitrogen-fixing symbiosis between *M. truncatula* and *Sinorhizobium meliloti* (Figure 6A). However, the interaction between MtSNARPs and *MtENOD40* in cytoplasm, as well as the functional role of MtRBP1 in cytoplasm require further studies.

### Long Non-Coding RNA-Coding Peptides

The long non-coding RNAs typically lack the protein-coding potential. However, some lncRNAs were found to code peptides. Soybean *ENOD40* could produce two small peptides of 12 and 24 amino acids long, both of which bind to a subunit of sucrose synthase nodulin 100 for modulating sucrose use in nitrogen-fixing nodules (Rohrig et al., 2002; Figure 6B). Another example of lncRNA-coding peptides is pri-miRNAs, which are primary transcripts of miRNAs. The *pri-miR171b* of *M. truncatula* and the *pri-miR165a* of *Arabidopsis* contain sORFs that code the peptides miPEP171b and miPEP165a, respectively (Lauressergues et al., 2015). Increasing the expression levels of miPEP171b and miPEP165a alters the accumulation of miR171b and miR165a, reduces the lateral root development, and promotes the primary root growth (Lauressergues et al., 2015). A similar mechanism has been reported in other life processes, such as those involved in miPEP858a encoded by *pri*-*miR858a* to modulate flavonoid biosynthesis in *Arabidopsis* (Sharma et al., 2020) and miPEP171d1 encoded by *pri-miR171d* to accumulate miR171d and regulate adventitious root formation in *Vitis vinifera* (Chen et al., 2020), indicating the universality of this mechanism in plants (Prasad et al., 2020).

## Future Directions

The finding of plant lncRNAs and their functional mechanisms is still in its early phase, and all the experimentally validated interaction pairs in plants are shown in Table 1. Although a large number of plant lncRNAs with unknown functions have been suggested by high-throughput sequencing, not all of them are functional. So far, only a small number of lncRNAs have been analyzed by the low-throughput biochemical and molecular biology functional assays. In fact, only 506 functional lncRNAs and 41 interactions of lncRNAs biomolecules in 56 plant species have been recorded in the recent update of the database for EVLncRNAs (https://www.sdklab-biophysics-dzu.net/EVLncRNAs2/) (Zhou et al., 2020). These data are only a tiny fraction of what can be possibly found. More experiments are needed to expand our knowledge in plant lncRNAs. Many questions that are to be addressed are as follows:

- What are the patterns in the secondary structure of lncRNAs in recognizing interacting molecules?
- What are the key sequence or structural factors that affect lncRNA functions?
- What is the degradation mechanism of lncRNAs in organisms?

These questions highlight the importance of RNA structures in providing functional clues. Recent advances in deep learning of protein structure prediction (Senior et al., 2020) and RNA secondary structure (Singh et al., 2019) highlight the importance of huge data in structure and function prediction. However, unlike proteins, the RNA structural and functional data remain scarce. As a result, the power of deep learning is quite limited until more data are available.

## Conclusion

As illustrated in Figure 2, functional lncRNAs identified so far respond to the plant development signals, which act as regulators in plant growth and development in association with other macromolecules. LncRNAs in plants can respond to a variety of signals, including light (Wang Y. et al., 2014), phytohormones (Ariel et al., 2020), salt (Qin et al., 2017), temperature (Wunderlich et al., 2014; Kindgren et al., 2018), deficiency of nutritions such as phosphorus (Franco-Zorrilla et al., 2007; Zhang et al., 2019), and biotic stresses such as pathogen infection (Hou et al., 2020).

Currently, plant lncRNAs were shown to function through the formation of eTM with miRNA to regulate the expression of miRNA target genes indirectly (Figure 3A), acting as decoys to hijack nucleic acids or proteins to prevent their functions (Figures 3A, 5B), the recruitment of modified proteins to remodel chromatin status at the epigenetic level (Figures 4A, 5A), or the linkage of macromolecules to form functional complexes (Figures 5D,E). Similar mechanisms of interaction between plant lncRNA and other macromolecules can be found in eukaryotes such as humans, mammals, insects, and even fungi (Quinn and Chang, 2016; Golicz et al., 2018; Kazimierczyk et al., 2020), further suggesting the conservation of biological evolution on the earth. It should be noted that lncRNA is a single-stranded RNA that can recognize the complementary RNA or DNA sequences. Although some tools have been developed to predict the potential complementary nucleic acid molecules of lncRNA, the low abundance of lncRNAs makes it necessary to be certain about their biological effects by conducting the low-throughput experiments.

Taken together, understanding the functions of plant lncRNAs is just at the beginning stage. Model plants such as *A. thaliana* and *O. sativa* are the most studied plants (Figure 1). Significantly more studies are needed to cover the ocean of lncRNAs with unknown structures and functions. These fundamental studies are practically important because these lncRNAs are implicated in plant yield and quality and disease resistance. According to the current experimental identification technology, such as CRISPR/Cas9, RNAi, and loss and gain functions, the rate of functional lncRNA verified by the biological experiments is ~600 per year (Zhou et al., 2020). It will be a long journey to fully understand the role of the massive amount of potential functional lncRNAs in the living organisms. Moreover, the growth of new high-throughput lncRNAs is accelerating. Thus, it is time to shed more light on the lncRNA “dark matter” to discover new “stars.”

## Author Contributions

QC conceived the topic of the article. QC, KL, RY, PH, and ZC wrote the manuscript. BZ, YZ, and JW reviewed and revised the manuscript. All authors have directly contributed to the work and also read and approved the final published version.

## Conflict of Interest

The authors declare that the research was conducted in the absence of any commercial or financial relationships that could be construed as a potential conflict of interest.
